# Palliative Care in Older People with Multimorbidities: A Scoping Review on the Palliative Care Needs of Patients, Carers, and Health Professionals

**DOI:** 10.3390/ijerph19063195

**Published:** 2022-03-08

**Authors:** Laura Llop-Medina, Yu Fu, Jorge Garcés-Ferrer, Ascensión Doñate-Martínez

**Affiliations:** 1 Polibienestar Research Institute, University of Valencia, 46022 Valencia, Spain; laura.llop@uv.es (L.L.-M.); jordi.garces@uv.es (J.G.-F.); 2 Population Health Sciences Institute, Newcastle University, Newcastle NE2 4AX, UK; yu.fu@newcastle.ac.uk

**Keywords:** palliative care needs, older patients, multimorbidities, caregivers, health professionals

## Abstract

Although numerous studies have been conducted previously on the needs of cancer patients at the end of their life, there is a lack of studies focused on older patients with non-oncological complex chronic multipathologies. Examining these needs would help to gain a greater understanding of the profile of this specific population within the palliative care (PC) pathway and how the health and care systems can address them. The aim of this review was to identify the needs influencing PC among older patients with multimorbidities, their relatives or informal caregivers, and the health professionals who provide care for these patients. A scoping literature review guided by the Systematic Reviews and Meta-Analyses extension for Scoping Reviews (PRISMA-ScR) checklist was carried out with literature searched in the Medline, Embase, CINAHL, WoS, Cochrane Library, PsycINFO, and Scopus databases from 2009 to 2022. Eighty-one studies were included, demonstrating a great variety of unaddressed needs for PC among chronic older patients and the complexity in detecting those needs and how to refer them to PC pathways. This review also suggested a scarcity of tools and limited pathways for professionals to satisfy their needs for these patients and their families, who often felt ignored by the system. Substantial changes will be needed in health and care systems at the institutional level, providing more specialized PC environments and systematizing PC processes.

## 1. Introduction

The palliative care (PC) concept has undergone changes and evolution in related conceptual and strategic approaches, including their use in clinical practice, in recent years. Conceptually, in 1990, the World Health Organization (WHO) adopted the definition proposed by the European Association for Palliative Care (EAPC) as the total active care of patients whose disease does not respond to curative treatment. The control of pain and other symptoms, as well as psychological, social, and spiritual problems, is paramount. The WHO subsequently expanded this definition, currently taking the form of: “An approach that improves the quality of life of patients and families facing life-threatening diseases, preventing and mitigating suffering through early identification, pain assessment and treatment, and other physical, psychosocial and spiritual problems” [1].

According to this definition, the delivery of PC should be guided by the improvement in patients’ and their families’ quality of life. Additionally, PC aims at facing needs associated with life-threatening conditions and trying to prevent and relieve suffering through several actions in the continuum of care: early and timely identification, adequate assessment and treatment of multi-domain symptomatologies. PC is applicable at the onset of the disease, alongside other curative therapies. This integration of PC has been developed mostly around the oncology area as most of the PC protocols, programmes and units are more focused and addressed to patients with cancer. However, the rate of older adults (60 and over) in need of PC with non-oncological diseases, such as Chronic Obstructive Pulmonary Disease (COPD), diabetes, cardiovascular disease and renal diseases [2] among others, is higher than those with cancer [3], and additionally, when they are admitted to PC units they tend to be closer to death than those patients with cancer [4]. Several systematic reviews have reported that patients with advanced cancer experience improved quality of life and symptom intensity with early PC interventions compared to those who only received cancer care alone [5,6]. There are also numerous studies that have been previously conducted on the needs of cancer patients at the end of their life, such as Bandieri [7], Haun [5], and Wang [8], however there is a lack of studies focusing on older patients with non-oncological complex chronic multipathologies, therefore, this study has focused on the needs of this profile of patients.

Chronic diseases represent around 70% of deaths worldwide [9]. The increased incidence, prevalence, and mortality of chronic diseases and multimorbidities, defined as the presence of two or more long-term health conditions [10], place a significant challenge on PC resources and a burden on health policies and practices [11]. As an increase in the rate of older people with complex chronic diseases is expected over the next 25 years, early identification of PC needs among older patients with comorbidities is becoming an important concern to health systems [12] in order to provide comprehensive care that meets the needs of both patients and family caregivers, considered by the WHO as an indivisible unit in the provision of PC. Ensuring that family caregivers’ needs are appropriately assessed is one of the top quality markers for end-of-life care [13], due to them being critical to the patients quality of life. Thus, the aim of this scoping review was to identify the PC needs of patients with multimorbidities (excluding cancer), their family caregivers and the professionals that work with these patients to obtain a global vision of the actors involved in the provision of PC. This study provides a comprehensive analysis of the needs and concerns around PC provision for non-oncological chronic conditions from a broad approach, joining perspectives of three target groups.

## 2. Materials and Methods

This scoping review used the framework of Arksey and O’Malley [14], which comprises five stages: identifying the research question; identifying relevant studies; study selection; charting the data; and collating, summarizing, and reporting the results. The Systematic Reviews and Meta-Analyses extension for Scoping Reviews (PRISMA-ScR) checklist was used to guide the conduct of this review, which contains 20 essential reporting items and two optional items to include when completing a scoping review [15]. The protocol of this scoping review has not been registered or published.

### 2.1. Identifying Relevant Studies

Thorough electronic searches were carried out in the Medline, Embase, Cumulative Index to Nursing and Allied Health Literature (CINAHL), Web of Science, Cochrane Library, PsycINFO, and Scopus databases covering the period January 2009 to February 2022. A range of keywords and subject headings indicating PC needs, older people’s needs, patients, families, non-formal caregivers, and health care professionals were used to maximize the retrieval of relevant studies. The specific questions addressed within this review were:

- What are the PC needs for older patients with multimorbidities?
- What are the PC needs for caregivers of older patients with multimorbidities?
- What are the needs influencing PC provision by health professionals for older patients with multimorbidities and their caregivers?

The final search strategies used in Scopus are shown below as an example:

- (((((palliative care [Title/Abstract]) AND patients [Title/Abstract]) AND needs [Title/Abstract]) OR preferences [Title/Abstract]) AND older [Title/Abstract] OR elderly [Title/Abstract]).
- ((palliative care [Title/Abstract]) OR palliative care unit AND health professionals[Title/Abstract] AND perceptions [Title/Abstract]) OR needs [Title/Abstract]) NOT patients [Title/Abstract]).
- ((palliative care [Title/Abstract]) AND families [Title/Abstract] OR caregiver [Title/Abstract]) AND needs [Title/Abstract]) OR perceptions [Title/Abstract]) AND elderly [Title/Abstract]) OR aged [Title/Abstract]).

### 2.2. Study Selection

The records identified from the electronic searches were imported into Endnote (Reuters, 2011) to avoid duplication of the screening process. Titles and abstracts were initially screened (by L.L.-M., A.D.-M., or Y.F.) to identify potentially eligible papers, and doubts were resolved between those reviewers. The full texts of potentially eligible papers were independently screened against eligibility (see Table 1) criteria by three reviewers (L.L.-M., A.D.-M., or Y.F.). No additional manual searching was carried out.

The papers included were peer-reviewed studies published between 2009 and 2022, in the English language, using any research design to report the needs or concerns in PC of older patients with multimorbidity, their families or carers, and health professionals in any healthcare setting.

The papers excluded were those about PC focused on cancer, published before 2009, involving patients younger than 60 years old, covering aspects of care and treatment not related to PC, lack of availability of full text, or full text available but paper written in any language other than English.

### 2.3. Charting the Data

Data were extracted employing a structured data extraction form that was tested in a sample of 5 studies by three members of the research team independently (L.L.-M., A.D.-M., or Y.F.) and were double-checked by a second reviewer (L.L.-M. or A.D.-M.). Data were extracted first by identifying the target groups involved (patients, caregivers, and health professionals). Secondly, data extraction was performed in a descriptive manner according to the following variables: Age group of patients, country of the study, source of data collection, type of service, care setting, main thematic findings, and reported limitations (see Supplementary Materials, Table S1).

### 2.4. Collating, Summarizing and Reporting the Results

Based on the data extracted, the included studies were classified according to the WHO’s PC definition. Final topics were agreed by three members of the research team. These final topics are presented in Table 2 below.

Due to the studies collected presenting a range of diverse methodologies, an exhaustive analysis of the quality of the studies was not carried out. However, the limitations of each of the studies included, if these were reported, are listed in the extraction table.

## 3. Results

**Figure 1:**
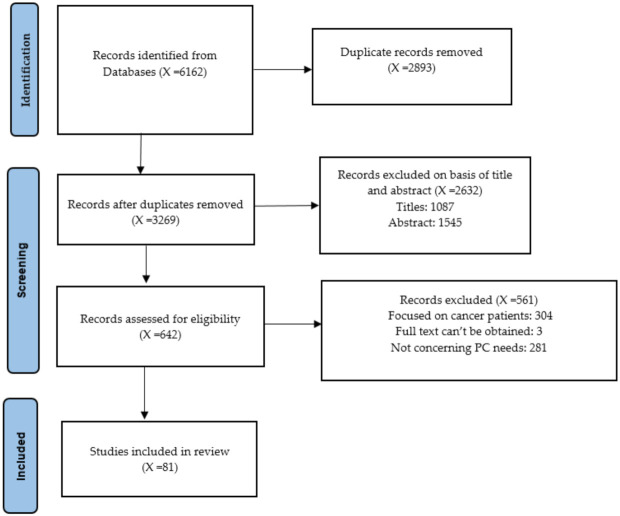
Study selection Prisma Flow Diagram.

A total of 6162 records were identified covering the time span of January 2009 to February 2022. From them, 642 records were selected for abstract review, identifying 81 final studies for full review. The process of the study selection is detailed in the PRISMA flow diagram (Figure 1).

Although most of the papers analysed focus on a single country (51), a great deal of heterogeneity has been detected in studies addressing two or more countries. Next is detailed the distribution of studies by country. Most of the studies included were conducted in the UK [16,17,18,19,20,21,22,23,24,25,26,27,28,29,30,31,32,33,34,35], the USA [36,37,38,39,40,41,42,43,44], Australia [45,46,47,48,49,50,51,52,53], Canada [54,55,56,57,58,59,60], and Germany [61,62,63,64,65,66]. Some studies were performed in two or more countries: Belgium, Netherlands, UK, Germany, Hungary [67]: North America, Australia [68], UK, Ireland, USA [69], USA, UK, Australia, Canada, Belgium, Germany, Hong Kong, Japan, Malaysia, Singapore [70], USA, UK, Sweden, Netherlands, Spain, Canada, Ireland, Australia, New Zealand [71], South Africa, Kenya, South Korea, United States, Canada, UK, Belgium, Finland, Poland [72], Australia, New Zealand, UK [73], Canada, USA, UK [74]. The rest were in Belgium [75,76], Sweden [77,78,79], Brazil [80], China [81], Switzerland [82], the Netherlands [83,84], Norway [85], New Zealand [86], Denmark [87] and Hong Kong [88]. There were six studies with no information on the country/city where the research was performed [89,90,91,92,93,94,95,96].

Regarding the sources of data collection, the sources of data collection of all the studies analysed in the review are detailed in the following table (see Table 3).

Most of the studies (69.9%) focused on the perspective of one cohort: professionals (47.4%), relatives or caregivers (13.1%) and patients (9.4%), while the rest reported the needs of combined cohorts: patients, caregivers, and professionals (13.1%), on patients and caregivers (13.1%), and patients and professionals (3.9%).

Taking into account whether the studies can refer to several targets, some studies made reference to various targets, 31 addressed the perspective of patients, 31 addressed the perspective of caregivers and 53 analysed the perspective of professionals.

Studies included were undertaken in different health care settings: inpatient care (52.7%) [16,17,18,21,22,24,26,27,29,30,31,32,34,37,38,40,42,43,44,47,48,49,54,57,59,60,62,67,68,72,74,77,78,80,82,83,85,91,92,93] outpatient care (18.5%) [28,33,35,36,39,45,46,52,53,64,68,73,86,87], and combined inpatient and outpatient care (21.2%) [20,25,41,50,56,61,63,65,66,69,70,71,76,81,89,90]. In addition, five studies (6.7%) did not specify the care setting [19,23,58,75,94].

### 3.1. Patients’ Needs

Of the studies, 31 addressed the PC needs of multimorbid older patients only or together with other cohorts.

#### 3.1.1. Emotional/Mental Needs

Most of the needs reported were related to emotional and/or mental dimensions, such as social isolation, depression, anxiety, or feeling like a burden on families [18,28,34,39,41,69,71,91].

#### 3.1.2. Physical Needs

Patients reported that needs at the physical level caused less concern, as they were often considered covered by health providers through therapies or treatments. However, the most commonly reported physical needs were pain, fatigue, restlessness and agitation, and limitations in activities of daily living [18,34,39].

#### 3.1.3. Information Needs

A lack of enough information was also reported in several studies, highlighting the need for more information about PC and related resources [19,21,55,70,83,92], as well as the need for information about the progression and severity of their disease [63,70,71,89,95]. In line with information gaps, communication with health providers was also detected as an area for improvement, as patients consider it necessary to improve relationships and effective communication, for instance, to feel more supported in finding PC providers [39,40,41,49,71,74,89,95].

#### 3.1.4. Spiritual Needs

Some studies revealed the need for spiritual care [33,41,45,48,71,72,96], although these were approached from different perspectives, with some studies mentioning the need for spiritual attention without specifying more about it [33,48,71] and others referring to more specific aspects of this concept. In this sense, patients can feel distress if their religious wishes and values are not attended to, and being able to discuss spiritual beliefs was indicated as highly important to many patients [41]. Patients felt that clinicians lacked knowledge about cultural practices, such as rituals and religious aspects considered highly sensitive and necessary in end-of-life care [45,72,96]. In some cases, spiritual care was considered to be the possibility of talking to religious leaders, or as providing a safe space, communicating with sensitivity about spirituality, listening, and counselling [72].

#### 3.1.5. Other Needs

Other needs, such as specific cultural needs, were also mentioned, as well as clinicians’ lack of cultural awareness and the potential breadth of cultural practices, rituals, and other cultural patient’s needs [45].

The need for an adequate environment, with privacy and dignity, as well as the preference for receiving care in the same place and by the same clinicians in order to become comfortable with the environment and to minimize distress, especially in cases of patients with dementia, were also reported [33,48,74,83,94]. Moreover, the appointment of a key worker acting as a point of contact for the patient and family was considered relevant for continuity of care, avoiding confusion, and maintaining continuous contact [20].

### 3.2. Caregivers’ Needs

Thirty-one of the studies addressed relatives’ or informal caregivers’ PC needs, together with other target groups in some cases.

#### 3.2.1. Emotional/Mental Needs

Similarly, to patients, some of the needs pointed out in the studies by caregivers were those related to emotional and/or mental aspects, such as sleep problems, stress, confinement, physical strain, or anxiety [44,57,95]. They also desired emotional support and educational courses regarding how to handle emergency situations, e.g., falls and psychosis or medications, and more information about disease progression and what to expect in the future [35,36,56,65].

#### 3.2.2. Physical Needs

Caregivers indicated the need for more respite services or personal outings to deal with fatigue and strain [35,57].

#### 3.2.3. Financial Needs

Other needs related to financial concerns were also mentioned in some of the studies. Some caregivers had to leave their paid employment to provide full-time care and use their savings or accumulate debt derived from paying for care for their family member, the costs associated with long-distance travel, medications, home care, accommodation, and the rental of equipment necessary for the care of the patient’s needs [36,39,44,49,58,74].

#### 3.2.4. Social Needs

Social needs were also detected in those studies related to the impact of caring on caregivers’ social life. These studies stressed the loss of and decrease in caregivers’ social contacts or the experience of isolation from friends, neighbours, and the community as a result of providing full-time care [18,36,44,58]. Social assistance needs to obtain resources or benefits, such as direct financial support or social services to alleviate the burden of care, were also mentioned [36,41,58].

#### 3.2.5. Other Needs

Some studies reported that family caregivers felt that the amount and type of information received about their patients’ health were inadequate and insufficient [36,51,55,65,91,95]. Some studies pointed out that caregivers consider care providers to be mainly focused on the medical aspects of care and that they tend to exclude the psychological, emotional, practical, and spiritual domains [41,58,72]. In this line, spiritual care was reportedly lacking, due to staff members’ lack of time and their lack of prioritization of this aspect of care; consequently, caregivers reported the need for spiritual care for patients and themselves and the need to have time to talk to clinicians about this [33,35,51,56,93]. Furthermore, more time and human connection with professionals, effective communication, and shared decision making were indicated as important [30,55,56,91].

Some studies reported that caregivers feel that they do not have enough information about PC services and how these can provide more comfortable care [20,29,56,68,84,91]. Regarding discussing end-of-life care preferences together with healthcare staff, some studies reported that family members would like to be more involved in medical decisions [74,93,95]. Caregivers often felt that medical staff, such as nurses or doctors, do not have enough time to listen to and discuss their relative’s condition with them, becoming a source of distress to families who feel under pressure to make the right choices [33]. One study emphasized concerns about combining work with caring for their family member, highlighting the need for remote working and flexibility in working hours [84]

Lastly, other studies highlighted bereavement support as an important need for caregivers. Carers described the continued need for support in the period soon after death [33,56,84], and remarked that palliative care provision should be extended to support family carers [20,35,56,58].

### 3.3. Professionals’ Needs

Fifty-three studies focused on professionals only, or together with other target groups, to approach PC. Two main domains were found: The needs and concerns of professionals in the provision of PC for multimorbid older patients and needs related to specific training in PC provision.

#### 3.3.1. Needs in PC Provision

On the one hand, professionals identified barriers to providing effective PC to this group of patients. In some studies, professionals pointed out that patients with non-malignant disease were less likely to be referred to PC services due to the historical link between cancer and palliative care [24,25,66]. In other studies, professionals highlighted that patients suffering from non-cancer diseases often receive inadequate care, with poor communication between the different services that care for them, and also shared that there are fewer specific services for these patients compared to for cancer patients [61,83]. A lack of staff to provide sufficient care to patients, resulting in little time to properly address PC needs, was highlighted by some studies [48,79,80,81].

(a) Referral to PC

Often, multimorbid patients are referred to PC units in the terminal stages of their disease; thus, professionals consider it necessary to initiate these referrals earlier to promote continuous care [78,85], especially with older patients [61], patients with Chronic Heart Failure (CHF) and COPD [75,76], and patients with conditions other than cancer [25]. Moreover, professionals do not always know who has the responsibility to care for CCC patients in need of PC, and often the roles are not defined, which causes fear of starting conversations about palliative care with the family or the patient [26,60,66,90].

In this sense, some of the studies showed a lack of homogeneous referral criteria, protocols, or pathways to initiate PC services [31,52,67,76]. Additionally, professionals pointed out that there are barriers to referring PC patients and interspecialty dialogue, with a lack of communication between different specialists resulting in professionals only partially knowing the pathologies that can occur in patients and preventing or delaying referral to a PC itinerary [46].

Moreover, in some studies, professionals expressed that there is complexity in prescription and treatment approaches due to the impact of the complex comorbidity profiles of multimorbid patients, such as the effects of drugs in older populations (unforeseeable interactions or side effects) [61,90].

(b) Comprehensive and continuous care in PC

Professionals considered that a holistic approach to patient care is crucial, starting when curative treatment is no longer realistic, rather than focusing only on physical symptoms [16,21,76,79,88]. The necessity of going beyond the management of pain was underlined, as patients’ and their relatives’ non-physical needs, such as spiritual and emotional needs, were considered to need further support [16,76].

Some studies also pointed out that professionals need more time and continuity in the attention given to their patients, having time to talk calmly with patients and their families about PC decisions and needs [31,62,81,82,87]. The pressure of having less time was indicated as causing a feeling of not being able to talk with or attend to patients in a comprehensive way [62,82], and lack of continuity was pointed out as a major threat to PC attention, especially for people with severe dementia [32,79,85]. Two studies remarked on the need to take into consideration the biography of each patient, and reported that practitioners considered that they only overviewed fragments of care among older patients, which makes the provision of individualized care adapted to patients’ needs difficult [52,61].

Some studies also remarked that adequate time is not available for professionals to be able to start conversations with relatives and patients about care planning and advanced directives [66,76]. Two studies stressed that professionals lack knowledge and belief in the role of advanced plans, since many changes can take place in the medium and long term [27,85]. It was also pointed out that there is a need for an objective measure to identify caregivers at risk of poor bereavement [50,53] and dedicated staff to take responsibility for bereavement care [50,57]. Furthermore, a need for formal assessments of bereavement instead of using only observations, intuitions, or informal conversations was highlighted [50].

A proactive multidisciplinary approach (combining clinical psychologists, social workers, and psychiatrists) and interprofessional collaboration [24,66,71,78] were described as important in PC to increase CCC patients’ and their caregivers’ wellbeing [52,67,73,81,86,90]. Moreover, new specialized structures for PC geriatric patients were considered necessary to be able to address the specific needs of patients with multiple needs [61].

#### 3.3.2. Training Needs

On the contrary, several training needs were detected. Special reference was made to specialized training in PC, highlighting that professionals may feel they have a lack of skills for good communication with patients [89], the need for additional palliative and end-of-life care education [19,38,80,86], more awareness and understanding of PC, as well as further training on how to identify patients’ needs and understanding of end-of-life care [66,81]. Additionally, several studies highlighted the lack of knowledge of the palliative needs of different groups of patients, such as those with COPD [87], severe dementia [18,68], disabilities [31], or complex comorbidity profiles [46]. Some studies stated the need to move toward early PC conception, as many professionals recognized that their perception of PC was associated with care during the last days of life [22,29,47]. Furthermore, one study highlighted the need for advanced training in early PC [75].

Some studies also pointed out professionals needing emotional support to prevent burnout and delineating emotional and professional boundaries [43]. One study highlighted the need for self-care in coping with death and dealing with professional grief [31], as some professionals felt that their grief is inappropriate or that they do not have time to express it [27].

In this line also, healthcare professionals remarked on the importance of spiritual care and having the skills to address it [16,73]. However, it was pointed out that professionals felt fear of being unable to resolve spiritual problems and experienced difficulties in communication about spiritual needs due to a lack of knowledge around such issues [17,96]. Additionally, they highlighted that assessment tools for spiritual needs are not taught during the received training [17,96]. Moreover, practitioners and nurses indicated a desire for support to address patients’ spiritual needs from other professionals, such as social workers, clinical/counselling psychologists, chaplains/spiritual care professionals, other alternative therapy professionals, and psychiatric professionals [81].

## 4. Discussion

This review contributes to a greater understanding of the needs of three cohorts involved in PC: older patients with non-malignant diseases, their family members or caregivers, and the health professionals delivering care for them. Integration of needs reported by them enabled a more comprehensive understanding of PC needs than those that focused on a single cohort only. Moreover, this review synthesized the needs for these three groups, which are very complex, diverse, and heterogeneous.

The studies included presented the support needed by patients related to their emotional/mental health, such as symptoms or experiences related to anxiety and depression. Supporting needs to address their clinical symptoms and to maintain their daily living activities was highlighted, and some studies also discussed patients’ concerns about the potential burden that their families or caregivers may experience as a consequence of covering their care needs. The needs identified in caregivers mirrored those identified at the patient level, but emphasized bereavement support, need of more information and human connection with professional’s involvement in decision making and financial support to care for both patients and themselves. It is worth highlighting that patients and caregivers noticed a lack of support in their psychosocial and spiritual needs, while considering their physical needs as being well-addressed.

In line with the results reported by Mathews et al. [97], where the integration of specialized palliative care with oncology patients was analysed, health professionals agreed that integrated PC services are the desired care models to facilitate integration and coordination of care using an interdisciplinary approach [74,77,78]. This study also highlighted the need to allocate more personnel, time, and training to assessing and addressing the needs of oncological patients and their families or caregivers, as identified in our review. Our review also highlighted the need for health professionals’ training in palliative care across all services (primary care, ICU, residences, emergencies, home hospitalization units), especially for providing care to non-cancer patients, as well as the need for itineraries and defined responsibilities in PC and more time to attend patients holistically and discuss with family members their needs and wishes [16,35,61,76].

The follow-up of grief for caregivers was highlighted as an urgent issue, with professionals considering it neglected [47,50,53]. To substantially improve the holistic attention provided for caregivers and relatives of CCC patients, it is essential to establish mechanisms to identify people at risk of complex grief and to have time and qualified personnel to deal with this issue within the context of PC.

Both patients and caregivers highlighted the need for spiritual care [33,35,39,73], but professionals felt a lack of knowledge and skills around related issues [16,17,37]. Moreover, there is no unanimity about what spiritual care means [21,26,61,96]. This is in line with the results of Gijsberts et al. [98], and considering the positive effects of spiritual care reported on patients, this dimension of care needs to be more visible.

This scoping review followed a systematic method and examined a wide range of studies that included three different perspectives (patients, relatives/informal caregiver, and healthcare professionals). This approach allowed for a complete and holistic view of the experiences and needs of older patients with multimorbidities at PC pathways. However, there are some limitations in this review. First, the study only included studies published in the English language; thus, some relevant studies could have been excluded. Moreover, we did not perform manual searching of key journals, and grey literature was not included, as we carried out searches in seven commonly used databases. This review it is not intended to establish definitive conclusions about all the needs of the target groups, as it is a scoping review and the intention of the study has been to make a first approach to them, since the research questions were very broad. We sought to map the needs detected in the literature for the three target groups with the aim of being able to deepen them in future studies. As this is a scoping review, no risk of bias assessment was conducted. In addition, we can only extract results of the studies with available data, acknowledging the limitations of the analysed studies, including issues such as the exclusion of some countries if studies were not published in English language. However, we consider there to be sufficient accuracy and consistency of the results analysed in this scoping review for it to be useful to guide future research.

## 5. Conclusions

This review provided evidence highlighting that substantial changes are needed in health and care systems at the institutional level to provide more specialized PC environments and systematized PC processes for multimorbid older patients. It is vital to understand and address the needs of multimorbid older patients and their caregivers given that the number of these patients is growing, which may challenge current healthcare systems. Regarding clinical practice, the identification of older chronic patients in need of PC will allow healthcare professionals to plan care and pathways in advance, and to reduce unnecessary admissions to emergency departments. Therefore, a systematic assessment of needs through appropriate tools in clinical practice is necessary to enable the development of an individualized integrated PC model for patients with complex PC needs. These findings can guide policymakers in increasing investment in specialized PC services focused on multimorbid patients to support more efficient use of available resources for healthcare professionals in their routine practice. For future studies, it is recommended to take into consideration patients comorbidities, since their needs vary accordingly, as well as the particular requirements around PC of those older people with COVID-19.

## Figures and Tables

**Table 1:** Inclusion and exclusion criteria.

Criterion	Inclusion	Exclusion
Period	1 January 2009 until 7 February 2022	Published before 2009
Language	English	Any other languages
Type of studies	Qualitative, quantitative, and mixed method studies published in peer-reviewed journals	Letters, comments, conference abstracts, editorials, doctoral thesis
Type of participants	Older patients with multimorbidity (presence of two or more long-term health conditions) Caregivers of older patients with multimorbidity Health professionals in any PC healthcare setting	Cancer patients Patients who do not have multimorbidity (presence of two or more long-term health conditions) Patients under 60 years of age
Type of outcomes	Concerning Palliative care needs	Not concerning palliative care needs

**Table 2:** Final topics according to the WHO’s PC definition.

Patients	Caregivers	Professionals
Emotional/mental needs Physical needs Information needs Spiritual needs Other needs	Emotional/mental needs Physical needs Social needs Financial needs Other needs	PC provision Training needs

**Table 3:** Sources of data collection.

Sources of Data	References
Individual Interviews	[18,26,27,32,44,46,48,58,59,62,63,76,77,80,82,90,92]
Focus groups	[17,18,25,48,50,51,57,61,64,72,75,78,86]
Individual interview and focus group or similar	[22,24,28,33,35,36,42,47,49,53,85,87]
Individual interview and questionnaire or survey	[65,81]
Survey or questionnaire	[30,34,37,38,39,52,56,66,69,73,83]
Reviews	[34,62,64,65,68,80,82,84]
Individual interview, focus group or similar and survey	[20,31,54,60]
Individual interview focus group or similar and survey and RTC	[21]
Individual interview, ethnographic observation	[29,41]
Non-participant observation, semi-structured interviews, focus groups and a co-design event	[19]
Individual interview, retrospective audit of existing hospital databases	[45]
Q-methodology (combination of qualitative and quantitative techniques)	[94]

## Data Availability

No applicable.

## Supplementary Materials

The following supporting information can be downloaded at: https://www.mdpi.com/article/10.3390/ijerph19063195/s1, Table S1: Data extraction table.
