# Supplementary material for: Palliative Care in Older People with Multimorbidities: A Scoping Review on the Palliative Care Needs of Patients, Carers, and Health Professionals
Source: Int J Environ Res Public Health. 2022 Mar 8;19(6):3195. doi: 10.3390/ijerph19063195 (PMC8954932; doi:10.3390/ijerph19063195)
Supplement: Supplementary file 1 [file ijerph-19-03195-s001.zip › ijerph-1561412-supplementary.pdf]

S1: Data Extraction table

	Study	Target	Source of data collection	Age group of patients	Main thematic findings	Reported limitations
**16**	O'Brien et al 2019	Professionals	Individual interview	-	Participants emphasized the importance of identifying what matters to the individual, what is fundamental to them and what their priorities are at this stage of their lives.<br>The need to be aware of religious beliefs and how these can affect end-of-life care and preconceptions about spirituality and religion was detected, as many consider them to be the same thing.<br>Importance of recognizing that the environment, within which patients find themselves at the end of their lives, may be adding to their spiritual distress.<br>The value of allowing patients time to think was stressed.<br>Post course, there was realization that an essential aspect of providing holistic care was to ensure that participants addressed more than just the patient's physical well-being.	The study was retrospective with only one data collection time point; this must be considered as a potential limitation.
**17**	Abbas & Dein 2010	Professionals	Focus group or similar	-	Lack of knowledge about what spiritual needs mean.<br>Lack of training in spiritual needs assessment tools.<br>Difficulty detecting and treating spiritual needs or pain.<br>Tendency to consider spiritual needs as something related to religion and is considered something that belongs to the private sphere	The small sample size and the fact that this was a single site study mean that the findings cannot be generalized.
**18**	Bajwah et al 2013	Patients, Relatives or informal caregivers, Professionals	Individual interview	57-81	Scope of physical and psychosocial needs.<br>Professionals find a lack of skills and training to manage some symptoms.<br>All three groups had an impact on their social activities.<br>Patients and caregivers reveal significant changes in their social relationships causing isolation and loneliness.<br>The effects of the disease on caregivers are often not identified by professionals	Number of subjects interviewed is small<br>All subjects had a TLCO less than 40% indicating severe, if not terminal, disease. There can be marked heterogeneity in progression within the disease population.

* In all the studies both genders are represented.

						All patients and informal caregivers were recruited from specialist ILD centers in the United Kingdom.
**19**	Blackwell et al. 2017	Patients, Relatives or informal caregivers, Professionals	Non-participant observation, semi-structured interviews, focus groups and a co-design event	Only 'older patients' is mentioned	Patients and family members require more information and personalized attention. Professionals manifest a lack of training and experience in PC and on how to identify the needs of the patient. Lack of resources in the Emergency Department to provide palliative care.	Informal fact-finding exercise via email, this approach prevented probing questions and deep exploration
**20**	Bone et al 2016	Patients, Relatives or informal caregivers, Professionals	Individual interview, focus group or similar, questionnaire/s or survey	Elderly with frailty and hospital attendance	More information for decision-making or symptom management and bereavement support. Ensure older people are informed about to avoid anticipate plans and directives. Involve relatives in discussions about plan of care Identify carer's needs. Fast and easy access to specialist palliative care advice. Key worker assigned to patient for continuity of care.	Selection bias. The consensus survey was conducted in a single round
**21**	Buckingham et al 2015	Patients, Relatives or informal caregivers, Professionals	Individual interview, questionnaire/s or survey, RCT	Mean age 72.2	People highlighted the benefits derived from discussion about spiritual or psychological needs. Patients desired more information provision about resources. Professionals had a strong sense that existing services did not address the complex and long-term support needs of people with severe COPD. The uncertain prognosis meant that palliative care services were not adequate for these patients, as they are linked to the last months of life.	The findings of this unicentric study may not be representative of other contexts.
**22**	Davis et al 2014	Professionals	Individual interview, focus group or similar	-	Palliative care of patients with dementia was described as chaotic and disorganized, cancer patients receive specialized care while patients with dementia do not. The need for some element of systematization without too much rigidity was recognized. Palliative care for people with dementia was	Small sample (not all job roles involved in palliative care for people with dementia were included).

					described as fragmented and disconnected while a holistic model of care was desired.<br>Need of training in PC	
**23**	Fleming et al 2016	Patients, Relatives or informal caregivers	Focus group or similar	95-101 years	Patients expressed their desire not to prolong life with invasive treatments and to consult family members about patients' wishes. The main concerns of patients and family members regarding the end of life were to feel comfortable and stay at home avoiding hospital admissions and respecting the patient's wishes.	The lack of response potentially limits the validity of the research. An additional limitation was the study's inability to explore how older people's opinions might change over time, particularly as they approached death.
**24**	Gardiner et al<br>2011	Professionals	Individual interview, focus group or similar	-	Participants identified several barriers to the provision of palliative care for older people, such as; the role of the health professional is poorly defined in terms of providing palliative care, lack of psychosocial support available, patients with non-malignant diseases were less likely to be referred to PC services, partly due to the historical link between cancer and palliative care, lack of staff causing lack of time in care, sometimes doctors are too interventionist.	Not reported
**25**	Gott et al. 2011	Professionals	Focus group or similar	-	Time and resources to appropriately time transitions to palliative care was detected as a need<br>Improve communication between clinicians and patients about palliative care approaches.<br>Particular difficulties in communicating with patients with conditions other than cancer who were particularly likely to undergo a late transition to a palliative care approach.<br>Patients typically left hospital with a poor understanding of their diagnosis and prognosis.<br>Patients' preferences for place of death, and in particular dying at home, could not always be met for a range of reasons	Participants reported on their practice and that of their colleagues, therefore this was not directly observed. As all data collection was carried out in England, findings may not be generalizable to other countries.
**26**	Handley et al.<br>2013	Professionals	Individual interview	-	Lack of clarity about roles and responsibilities in providing end-of-life care.<br>Recognising deterioration was not easy and was a more	This study is limited in studying six care homes and associated primary care

					nuanced process than was openly discussed between primary healthcare and care home staff. Need of collaboration between care homes and primary healthcare staff and the need to discuss how residents and family members can be involved over time. There is a need for a pattern of working between health and care staff that can encourage review and discussion between multiple participants over sustained periods of time.	services in areas that may not be representative.
**27**	Livingstone et al 2012	Professionals	Individual interview	-	In general, staff felt uncomfortable discussing death-related issues with residents or family members. Some staff members felt that their role was to preserve life for as long as possible, sending people to the hospital, even against the wishes expressed by the family. They sometimes felt that they had no emotional support and that their pain was inappropriate or there was no time to express it. They also expressed a lack of knowledge in advanced plans or that perhaps these could be invalidated by residents' families.	The interviews were all conducted in a single nursing home in a large city, suggesting that the problems encountered may be different than elsewhere.
**28**	Lloyd et al 2016	Patients, Relatives or informal caregivers, Professionals	Individual interview, focus group or similar	Over the age of 75	Social and community support was identified as a source of well-being, the prominent fears were the loss of mobility and independence and being a burden for family members and caregivers. Refusal to speak openly about death was also detected.	Sample not include frail older people who may lack an informal carer or who have dementia.
**29**	Manson et al 2013	Patients, Relatives or informal caregivers, Professionals	Individual interview, ethnographic observation	-	Patients, caregivers and professionals identified problems related to communication difficulties, lack of information and collaboration in care transitions. Difficulties were detected in identifying patients in need of palliative care and were often identified when they were already in the last days of life. The professionals considered that they had only a partial understanding of the patients' long-term circumstances. In many cases, patients and professionals considered palliative	The variety of data sources occasionally caused difficult decisions about which areas to prioritize.

					care as care for situations where death is imminent. Caregivers reported that communication was inconsistent and had little information about the patient.	
**30**	Mayland et al. 2017	Relatives or informal caregivers	Questionnaire/s or survey	-	Participants expressed unmet information needs as they would have benefit in knowing more about what to expect as their family member was dying.<br>Nurses and doctors had not enough time to listen and discuss with family member's the patient's condition.<br>Relatives perceived that they were not involved in decision-making.	Not reported
**31**	McLaughlin et al 2014	Professionals	Individual interview, questionnaire/s or survey	-	Communication needs were identified between specialized palliative care services and disability care services, as well as end-of-life care training for people with intellectual disabilities, as services are often not adapted for people with disabilities. Likewise, in the intellectual disability services, continuous training needs were highlighted on how to manage physical symptoms and pain, end-of-life care and grief. Other learning needs were self-care in coping with death and professional grief. The need for better coordination and continuity of care was also highlighted.	Not reported
**32**	Lee et al 2015	Professionals	Individual interview	-	The professionals highlighted as important issues for the end-of-life care of people with dementia: knowing the person with dementia, interpreting non-verbal communication, listening to and respecting family caregivers and ensuring personal and physical comfort. They identified the following necessary interventions: ensuring continuity of care, listening to and respecting family caregivers in terms of leveraging existing knowledge of family members and providing post-bereavement support and managing the transition to shared care with staff. They identified barriers in elder care, such as; low salaries of care staff, high staff turnover and inadequate	Approach to the sampling of national and local experts was pragmatic and purposive and based on team meetings, snowball sampling and the identification of experts through searching.

					numbers of qualified staff. The need for specific palliative care for elderly patients was suggested and it was recognized that clinical and palliative experience has an important role to play in end-of-life care for people with dementia.	
**33**	Poole et al. 2012	Patients, Relatives or informal caregivers	Individual interview, focus group or similar	-	In general, both patients and caregivers emphasised the importance of remaining in their preferred place of care and ensuring comfort and minimising distress.<br>Being comfortable and pain-free.<br>Importance of psychosocial elements such as emotional comfort, compassion and spirituality.<br>well-trained staff, skilled in the delivery of individualized and person-centred care as well as they should have additional or specialist training<br>Patients desire for family to be with them at the end of life<br>Caregivers want to explicitly discussed wishes and preferences with their familiar (source of distress to families who felt under pressure to make the right choices)<br>Organizational barriers to coordinating good end-of-life care: navigating complex systems to access continuing healthcare funding, pressure from service providers to move their relative into institutional care and coordinating care providers from different services.<br>Some carers described the continued need for support in the co-ordination of care in the period soon after death	People with dementia were recruited from the community, and from one area of England, rather than through healthcare services. Therefore, the views of people with dementia living in residential care or people with dementia without family carers and regional variation were not captured.
**34**	Ryan et al. 2013	Patients	Questionnaire/s or survey	Mean age 70.85 years	Patients with PC need were more likely to identify higher levels of physical burden, including: weakness; tiredness; shortness of breath; restlessness and agitation.<br>Also they reported being bothered by a number of psychological burdens, including: anxiety; low mood; poor concentration and confusion.	Non-English speakers and those people who were unable to consent and did not have a close relative or friend who was available were excluded from the study. Small sample size used in the regression analysis

**35**	Waldron et al 2013	Relatives or informal caregivers	Individual interview, focus group or similar	-	Both participant groups stressed the need for the specialist knowledge of drugs in a palliative care setting, access to good nursing care and holistic support from social and health care professionals in terms of psychological, spiritual and emotional needs.<br>palliative care needs of carers:<br>palliative care provision should be extended to support family carers.<br>Practical help, information, emotional support, referral to appropriate agencies that might be of benefit and respite opportunities<br>Having contact with a PDNS from an early stage and access to bereavement care were regarded as valuable.<br>Support groups: Provision of care to people with non-malignant and malignant conditions<br>NHS-based participants: Scarcity of resources and funding in the current economic climate with heavy caseloads<br>Lack of knowledge and recognition among Primary Care Trusts' staff that clients with PD might need palliative care.<br>Lack of PD experience and training: The difficulty in defining the palliative care stage in clients with Parkinson's disease<br>Participants identified more resources, more knowledge, more understanding of PD, more information regarding advice and awareness.	Not reported
**36**	Boersma et al 2017	Relatives or informal caregivers	Individual interview, focus group or similar	-	Need of individual attention and support, of boundaries in their role as an informal caregiver and the importance of maintaining their own social lives and involvement in hobbies despite the illness.<br>Educational needs and helpful resources about disease progression, medications and their side effects, to respond to emergency situation sand useful resources.	Small sample size and thus may have missed issues important to many caregivers.

					Cognitive dysfunction was challenging and negatively impacted their lives. Financial concerns.	
**37**	Chang et al 2012	Professionals	Questionnaire/s or survey	-	Need of support for home visits in grief (for nurses) Importance of extending the bereavement care to recently deceased families and carers of their former palliative clients. A common misunderstanding that palliative care is only for the final phase of life. Difficulty addressing spiritual and cultural issues with patients and with the resident's family.	A higher response rate and sample size are desirable to increase the generalizability of results that is also limited by the inclusion of only one metropolitan health service administrative area.
**38**	Cimino et al. 2014	Professionals	Questionnaire/s or survey	-	Main learning needs and preferences were understanding topics such as principles of palliative care, pain assessment, pain management, and non-pain symptom management	Sample limitation (more than two-thirds of respondents were nurses or social workers)
**39**	Grudzen et al. 2010	Patients, Relatives or informal caregivers	Questionnaire/s or survey	65 years or over	A majority of patients exceeded cut-offs for financial need (help with insurance coverage, caregivers working to pay for medical costs caregivers missing work to provide care, or using savings or incurring debt to pay for medical expenses) Need of access to care (patients need help finding providers and/or clinics that could provide care for their particular illness). Patients needed help with household chores and had significant limitations in activities of daily living. Caregivers reported sleep problems, confinement, physical strain, and emotional or family problems related to caring for a loved one. Patients also manifest needs related to physical health, pain, fatigue, anxiety, and/or shortness of breath.	Patient sampling was not representative of all seriously ill, older adults.
**40**	Hall et al. 2011	Patients	Review	average age from 80.0 to 87.9 years	Often older people in resident care home are not referred to specialist hospice or palliative care services, their symptoms are poorly managed, they are frequently hospitalized unnecessarily, advance care planning and communication is	Heterogeneity of the three included studies that made it difficult to combine and compare their results, and the small

					often inadequate, and their families are dissatisfied with their care. Need of increase relationships between care homes and specialist palliative care services who can provide specialist support for residents with complex needs. Training to improve the care of residents. Development of multidisciplinary teams.	number of studies together with their risk of bias.
**41**	Karen Tallman et al 2012	Patients, Relatives or informal caregivers	Individual interview, longitudinal video-ethnographic Study	from 46 to 89	Patient, Family, and Caregiver Needs: Five major need themes central to the patient, family, and caregiver experience were identified: 1) sensitive, effective communication about advanced illness; 2) timely access to coordinated medical care; 3) respect for and honoring care decisions; 4) psychological, social, and spiritual needs; and 5) caregiver support.	
**42**	Kwak et al 2013	Professionals	Individual interview, focus group or similar	-	Professionals recognized that death and dying are taboo discussion topics. Participants' recommendations were to develop trust with elders over time; cultivate cultural knowledge and sensitivity to respect value orientations; promote designating a healthcare proxy; recognize and educate families and community leaders as critical partners in advance care planning and provide practical support as needed throughout the illness experience.	The views reported are based on a select sample of Care Managers team members working with frail, low-income elders in the Family Care programme, so the results may not be generalizable.
**43**	Perez et al. 2015	Professionals	Individual interview	-	Challenges associated to their work environment were detected, such as time pressures, unpredictable schedules and competing role demands. Also they detect challenges in addressing patients' mutable needs, managing family dynamics, and meeting patient and family demands and expectations. They found challenges in delineating emotional and professional boundaries. Need of training in mind-body skills, such as yoga, meditation, and education to combat the effects of stress and cognitive strategies to avoid negative thinking was detected.	This study represents the perspectives of clinicians from only one medical institution in Boston, findings are limited in generalizability and may not reflect the opinions of clinicians beyond this sample.

**44**	Thai et al 2015	Relatives or informal caregivers	Individual interview	65+	The caregivers who experienced a decline in their QoL identified the following reasons: they had less time for self (social and financial domains), their health declined (physical domain), and the emotional impact of the patient's illness took a toll on them (emotional domain).	Due to the small sample size, correlations cannot be established between the circumstances of care and how they affect quality of life
**45**	Bloomer et al 2018	Patients, Professionals	Individual interview, retrospective audit of existing hospital databases	over 65	Patients' and families' cultural needs before and after death were not always accommodated (influence by organizational constraints and inadequate communication)<br>Clinicians typically lacked cultural awareness and the potential breadth of cultural practices, rituals, and other needs considered essential to the provision of culturally sensitive EoL care.<br>Clinicians identified a need for further education to improve understanding of how to address cultural needs for older people and their families at the EoL.	The retrospective audit is limited by the quality of the evidence.<br>The study was conducted in one setting in Melbourne, Australia, findings may not be transferable across other settings or patient groups.
**46**	Broom et al. 2012	Professionals	Individual interview	-	Timely referral was beneficial to prepare patient and FGCs for the end of life, limit unnecessary treatment, and streamline care focused on quality of life. Some medical specialists lacked the knowledge, experience, or inclination to give due consideration to referral to palliative care understanding these derivations as a failure of curative treatment and causing a lower quality of life of the patient. The presence of palliative care clinicians was viewed as a significant facilitator of timely referral.	Not reported
**47**	Chang et al 2009	Professionals	Individual interview, focus group or similar	-	Difficulties evaluating people with advanced dementia because they are not verbally communicative which makes it difficult to manage pain, dysphagia, behavioral symptoms<br>Emotional difficulties derived from connecting with patients.<br>Lack of knowledge about palliative care and common misunderstanding that palliative care is only for the end-of-life	Small sample and random sampling may be a limitation

					phase.	
**48**	Fleming et al 2015	Patients, Relatives or informal caregivers, Professionals	Focus group or similar	-	Ensure a quiet and familiar environment favoring privacy for dementia patients and their families. Promote participation and contact with family members. Pay attention to spirituality. A key area of concern for practitioners was the lack of staff to provide sufficient care to their patients.	The views expressed in the focus groups may not be generalizable to the wider population of people with dementia, their family or professional careers as the sample is small and no steps were taken to attempt to make it representative.
**49**	Lewis et al 2019	Patients, Relatives or informal caregivers	Individual interview, focus group or similar	60 and over	Participants agreed that a good quality of life was the most important consideration in EOL care. They converged on the notion that a good quality of life is when an individual has control and can meet their own personal standards and expectations. For patients, attending appointments for care at the EOL could be challenging, with the difficulty of multiple visits to services that were not localized, compounded by poor mobility and the associated costs of transport.<br>Discussions about the EOL were identified as limited, lacking, too late and emotionally challenging, leading to a lack of sufficient understanding of each patient's wishes.<br>Caregiver participants described the impact of their loved one's life-limiting illness as putting their life on hold to care for another, making financial, career and personal compromises in order to do this.<br>Participants desire competent and caring health professionals to assure patient dignity and quality EOL care to patients and caregivers.	The majority of participants were females and caregivers and only Three FG were conducted.
**50**	Sealey et al. 2015	Professionals	Focus group or similar	-	There is no clear delineation as to which service is responsible for grief care. As patients move through the system and between various services, there is a chance that they and their caregivers will get lost between services, caregivers don't know where to turn when they need help or information.	The findings may not transfer to other locations with different palliative care service models although research shows that similar challenges to bereavement care exist in other developed countries

					Referral and timely interventions would reduce the risk of complex grief and PTSD. The professionals found it very challenging to evaluate the grief after the death of the patient, since there is usually no contact with relatives. A problem for the services was the amount of time that grief support could be offered to people, in addition in the services there are no staff dedicated to grief, this absence causes inconsistency in contact and follow-up. Bereavement former caregivers could be difficult to contact after the patient's death, if they did not respond to the call the follow-up was stopped. It was suggested that a measure be constructed that could be adapted for use in palliative care, prior to the patient's death, in order to assess the caregiver's risk of grief.	
**51**	Sneesby et al 2011	Relatives or informal caregivers	Focus group or similar	-	Communication issues including disclosure and consent (death and dying are taboo subjects they are rarely discussed). Attitudes towards medical treatment: tend to discontinue medication once symptoms have passed. Herbal remedies will often be used in conjunction with conventional medicine. Spiritual and religious issues: The main religions of Sudan are Christianity and Islam. The participants found great strength in their religious beliefs and considered it as an important need.	Not reported
**52**	Tan et al 2009	Professionals	Questionnaire/s or survey	-	Barriers such as poor communication and limited availability of afterhours services were identified. Some GPs indicated that they did not use the elements of the EPC because of the paperwork involved. it was also highlighted that it would be useful to have more information about what PC services are available in your area and what they can provide. Additional resources such as increased	The response rate was very low, particularly in the case of GPs

					access to PA consultants and PA nurses, out-of-hours patient data, and palliative care guidelines were suggested.	
**53**	Wiese et al 2012	Professionals	Individual interview, focus group or similar	-	Importance of providing care at the end of life in the place chosen by patients and relatives, in general they considered that the ideal place was the home but recognized that this is not always possible, sometimes they felt guilt when making the decision to transfer the patient to the hospital. They also expressed the importance of communication with external health services. They recognized the importance of paying attention in the post-death period and attending to the grieving process of family members.	The perspective obtained was only from the staff
**54**	You et al 2014	Patients, Relatives or informal caregivers	Individual interview, focus group or similar, Questionnaire/s or survey	Mean age 81.2 years	Patients and family members identified the same 5 elements as being the most important to address: preferences for care in the event of life-threatening illness, values, prognosis, fears or concerns, and questions about goals of care, these elements are infrequently discussed and that the concordance between preferred and prescribed care objectives is low.	The sample is not representative of populations that do not speak English or French as a first language and that may have different values related to end-of-life care
**55**	Simon et al 2013	Patients and caregivers	Individual interview	Mean age 80 years	Some patients and family members were reluctant to talk about advance directive processes, for fear of talking about the end of life. Others expressed a desire to plan the life-support treatments they wanted and which they didn't want in the future. Patients and family members manifested continuous contact with doctors as the most important thing and the absence of communication as a problem. They also identified the need for information on PCA in general and its benefits or difficulties. To start conversations about end-of-life care, patients felt that conversations should be initiated by their doctors but with those who are known and reliable to them.	The major limitation is the use of a semi qualitative tool (questionnaire). The sampling strategy also limited participants linguistically

**56**	Gallagher et al. 2013	Relatives or informal caregivers	Questionnaire/s or survey	-	Bereaved relatives felt that the patient/resident had an unmet need for personal care and emotional support and that their own emotional needs were not addressed adequately.<br>Lack of focusing on the goals of the patient and family, and provider's lack of insight into what a family can be expected to know about the dying process.<br>Need for families to be supported after the death of a patient or resident.	The sample size is too small to make causal arguments. Participants stated that their perceptions were affected by events before the 48-hour time period and admitted that this shaped their responses. Participants wanted to provide differential scores between nurses, doctors and support staff but the survey does not allow this.
**57**	MacLeod et al 2011	Relatives or informal caregivers	Focus group or similar	-	Caregivers reported unmet needs related to communication, emotional support and education. They found it difficult to navigate the health system and expressed problems dealing with fatigue and strain. The need for resources such as telephone support, respite services and referral mechanisms were noted as very important for family members.	Not reported
**58**	Maddalena et al 2018	Relatives or informal caregivers	Individual interview	-	Major themes identified included challenges navigating the health system, caregiver burden, perceptions of palliative care, symptom management, and decision-making.<br>Caregiver burdens are significant including physical, emotional, social, and economic dimensions. Some caregivers had to leave their paid employment to provide full-time care to their loved one.<br>There is a need to recognize and improve support for family caregivers and increase collaboration with nephrology and palliative care services from commencement of dialysis until death and into bereavement.<br>Care providers focusing primarily on the medical aspects of care and excluding the psychological, emotional, practical, and spiritual domains	Not reported

**59**	Mistry et al 2015	Professionals	Individual interview	-	It emerged as a crucial issue in EoL, addressing the non-physical needs of the patient including a holistic perspective of care attending to the psychosocial and spiritual needs of the patient and caregivers. They stressed the importance of communication and cohesion between health teams to provide coordinated care and respect the wishes of the patient and family by giving them the option to decide and participate in medical and EoL care decisions. To provide quality EoL care it is necessary that the family and the patient participate in the decision making and are informed of the options available to them.	Provider perspectives captured may not be representative of those in other countries
**60**	Sarti et al 2015	Professionals	Individual interview, focus group or similar, questionnaire/s or survey	-	A lack of expertise, and a lack of interest in gaining expertise, in palliative/end of life care (PC in the ICU) was detected.<br>Gaps in PC: not seen as part of practice; lack of interest; lack of general expertise in PC.<br>Gaps in conducting EOL discussions and determining goals of care: when to have EOL discussions; they are not always initiated; Where to have EOL discussions: emergency department, ICU or family physician's office; who should have EOL discussions: roles and responsibilities are poorly understood; lack of comfort<br>Resource gaps: lack of physician availability; lack of PC services	The provision of palliative care in critical care was assessed at a single community hospital and the study did not specifically obtain qualitative data from patients and families
**61**	Brueckner et al. 2009	Professionals	Focus group or similar	-	Move forward to an holistic approach to patient care (multidisciplinarity and cooperation) due to practitioners overviewed fragments of older patient care only.<br>Lack of specialist geriatric medicine in the outpatient sector in Germany.<br>Palliative care physicians get involved in the care of older palliative patients relatively late and the palliative care nurses	Not reported

					consider that general practitioners would also need palliative medicine and geriatric expertise.<br>Need of training in pain therapy and symptom control<br>Great Risk of unforeseeable interactions and side effects of drugs in elderly population.<br>Lack of financial resources and staff in PC.	
**62**	Geiger et al 2016	Professionals	Individual interview	-	The care of frail older patients represents a particular challenge for the GPs and is influenced by a variety of considerations, particularly the complexity and fragility of the overall situation, the expectations of patients and their families, the high organizational effort, and the prevailing contextual and individual circumstances.<br>The time and the effort required for these patients are generally high. Physical examinations and consultations take much longer than with younger patients, and home visits and terminal care for frail older patients are highly time-consuming.<br>never having sufficient time to provide the amount of care necessary within the practice routine (time and sufficient calm for discussions with patients and their families about their needs and questions at the end of life)<br>Regarding the collaboration with specialists, timely and sufficient communication exchange is vital, but often missing.	Not reported
**63**	Klindtworth 2015	Patients	Individual interview	≥70 years	Patients' identified functional constraints and changing conditions that lead to problems in activities of daily living as their main concerns. Often patients with heart failure do not have enough information about the condition and their prognosis in particular regarding the problems they will face at the end of life. In general, patients wanted to increase interaction with professionals and discuss aspects of the medical care they will receive.	Patients were only recruited in an inpatient setting at two geriatric hospitals and not in general practice or other outpatient settings, the tendency towards socially desirable statements must be considered as a limitation of this study.

**64**	Krug et al 2018	Professionals	Focus group or similar	-	Professionals detected challenges in communicating with patients, caregivers and within the professional network. There are difficulties in interpreting the needs of patients who cannot communicate verbally. Sometimes the professionals' own opinion of "appropriate" death influenced care decisions. They expressed the need to have a close relationship with caregivers but also problems with the overly demanding attitudes of some caregivers. In the professional network they stressed that structures are missing, not all services are available in all areas, they also detected lack of communication between professionals.	Due to the mixture of GPs and medical assistants in the focus group discussions, statements might be influenced by a possible social status gradient.
**65**	Weber et al. 2012	Relatives or informal caregivers	Individual interview, questionnaire/s or survey	-	Need of information. Caregivers wanted to be informed about the actual medical condition by the responsible physician Professionals' lack of time was strongly associated with the unfulfilled needs for emotional support, many relatives also lacked this type of care when physicians seemed to have enough time.<br>Emotional support. More than half of the respondents wanted the physicians to provide emotional support.	Questions regarding the relatives' needs and experiences were not tested for their psychometric properties in advance, thus partly limiting the quality of the data. Selection and recall bias.
**66**	Ziehm et al. 2016	Professionals	Questionnaire/s or survey	-	Interprofessional communication and communication with the patients and their relatives was seen as important for PC.<br>Type and scope of life-sustaining measures should be discussed with the patient.<br>PC should be aimed to care for the patient at home and emphasize that PC should be patient-centered.<br>PC should always include all people affected by the disease of the patient, i.e. relatives and health care professionals.<br>Cooperation of all healthcare professionals and medical disciplines are essential for PC of patients with CHF	Low response rate in the questionnaires, more than half of the participants were men, the generalization of the results is not indicated.

					Education for all healthcare professionals and collective education throughout all medical disciplines should be offered.<br>Healthcare professionals do not possess sufficient knowledge about the content and the possibilities of PC.<br>Death is still a taboo subject<br>No sufficient financial conditions for PC.<br>Time to initiate PC (In general, most participants thought that PC of CHF patients should be initiated earlier than it is actually practiced)	
**67**	Herder-van der Eerden et al<br>2018	Professionals	Focus group or similar	-	Need for a proactive approach to multidisciplinary palliative care including an anticipated holistic assessment of the patient's current and future needs and desires, as well as multidisciplinary collaboration among all professionals involved in patient care.<br>Need for standardized criteria, protocols, or pathways, particularly for patients suffering from diseases other than cancer (COPD, heart failure, and other chronic non-malignant diseases). In these cases, referrals to PC often occur too late.<br>Problems with funding and availability of trained personnel.	Due to the complex international context, it was difficult to fully achieve an iterative process of simultaneous data collection and analysis, that would be considered as a limitation.
**68**	Goodman et al.<br>2010	Patients, Relatives or informal caregivers, Professionals	Review	65 years and older	Have structured time to communication, addressing issues of resident comfort, avoidance of tube feeding and care in a specialist unit.<br>Health professionals are not skilled at recognizing the end stages of dementia.<br>Families need assistance to process difficult and painful emotions, to understand the disease trajectory, to appreciate that some decisions might impede a natural death and to grasp the available comfort and palliative care options.	The absence of a shared understanding of how end of life is recognized may have resulted in some documents being excluded from the review

**69**	Higginson et al 2017	Patients	Questionnaire/s or survey	65 years and older	Patients stressed that they would like to make decisions about the care they will receive, preferred to receive the treatments and face the last period of life at home and if not possible in the hospital within a palliative unit. In some cases, extending their life would be more important than its quality Home was consistently the most preferred place to dead and hospital the least preferred place, with no differences between countries adjusting for age, gender. Non cancer was associated with a preference for in-patient hospice and palliative care however this usually being little available	People with cognitive impairment were excluded this makes the results not generalizable to all older people.
**70**	Ke et al. 2017	Patients	Review	Older people	Older people's perspectives and experiences of Advanced Care Planning were varied and often conflicted; cultural differences amplify variances among older people.<br>Participants remarked that it was necessary to have time to talk about EoL care, they wanted to have a private and quiet environment to discuss their concerns and make decisions. Feeling they had little information about end-of-life care and often didn't understand terminology, many of them expected healthcare professionals to start conversations about advance care planning. They also expressed concerns about the fear of being a burden to their relatives.	The review included only articles in English and the search was conducted in four databases, some relevant studies could be excluded. The age of the participants in the included articles was from 50 years to more than 90 years.
**71**	Low et al. 2011	Patients	Review	Mean ages from 67 to 92 years across all studies	Patients identified a good patient-professional and interprofessional communication as an important need. They desire an appointment of a designated health professional who would assume responsibility for patient assessment and care coordination.<br>They also stressed the need for recognition of their physical, psychosocial and spiritual needs that go beyond those related to their state of heart failure.	Lack of descriptive data makes it difficult to compare experiences across different countries and health care systems.

**72**	Selman et al 2018	Patients, Relatives or informal caregivers	Focus group or similar	Patients median age 62 years,	Five themes were identified: patients' and caregivers' spiritual concerns, understanding of spirituality and its role in illness, views and experiences of spiritual care, preferences regarding spiritual care, and research priorities.<br>Patients and caregivers reported a lack of that spiritual care and perceived insufficient staff time as a barrier to the provision of good spiritual care. Human connection was perceived as a prerequisite to providing effective spiritual care They also emphasize seeing spiritual care as an integral part of care and the staff should avoid categorizing or stereotyping. Patients wanted conversations about spirituality and spiritual needs to be initiated with sensitivity, they also wanted information about the availability of spiritual care and self-care practices, and to improve public discussion about death and dying.	A limitation of this study is that did not conducted back translation to check the validity of the translations.
**73**	Smallwood et al 2018	Professionals	Questionnaire/s or survey	-	doctors recommended referring people with advanced COPD to specialist palliative care, mainly for access to: psychosocial and spiritual care (105, 59.3%), carer support (104, 58.5%), and end-of-life care (94, 53.1%).<br>Themes highlighted in open responses included: inadequate, fragmented models of care, with limited collaboration or support from palliative care services.	The response rate in this study, whilst similar to other online surveys of physicians was low, thus limiting the generalizability of our findings.
**74**	Virdun et al. 2015	Patients, Relatives or informal caregivers	Review	-	Effective communication and shared decision-making was highlighted as of great importance to both patients and families. For the patients the most important thing was; honest communication, the ability to prepare for the end of life, ensure someone's availability to listen and be aware of what to expect from their physical condition. In relation to shared decision-making, patients specifically noted the importance of appropriate testing and treatment, not receiving life support	A single author examined the titles and abstracts and undertook data extraction for included studies. Only descriptive data were reported and therefore should be seen as informative rather than definitive. Additionally, the focus on purely quantitative data allows discrete categorical data. The sample involved in this review is biased towards Western

					when there was little hope of recovery, and having the opportunity to nominate their preferred decision maker. Families stated the importance of feeling supported in decision-making and having a sense of control over their loved one's care. Both patients and families noted the importance of comprehensive care, specifically in relation to effective discharge and family planning to ensure that the patient died in the desired location. Patients noted that the preservation of dignity was extremely important. Ensuring that one is not a physical or emotional burden was classified as very important by patients. Two major American studies pointed to the importance of financial issues in relation to end-of-life care.	developed world culture, White adults, predominantly older patients and female family caregivers (adult children or spouses).
**75**	Scheerens et al 2018	Professionals	Focus group or similar	-	Needs were related to the unpredictability of COPD, a lack of disease insight and resistance towards care of the patient, lack of cooperation and experience with palliative home care (PHC) for professional caregivers,<br>lack of education about early integrated PHC, insufficient continuity of care from hospital to home, and lack of communication<br>about PHC between professional caregivers and with end-stage COPD patients.	Not all participants met the predefined inclusion criteria for the study. Lack of knowledge of other professional caregivers involved in the care of patients with end-stage COPD (pulmonologists and physiotherapists
**76**	Siouta et al 2018	Professionals	Individual interview	-	Provision of a holistic approach is crucial for the care of patients with CHF and COPD.<br>Most mentioned that PC should start when the curative treatment is no longer realistic.<br>PC is implemented frequently at the end of life but that it should be ideally initiated earlier in the disease trajectory for the benefit of the patients.<br>Lack of time as a potential barrier for not assessing the goals (wishes of the patients) more regularly in reality.<br>Advance care planning (ACP) is central/mandatory to	This study opted for a total population purposeful sampling, but only a fraction of the population agreed to participate.

					CHF/COPD patients.<br>PC team is not involved in the treatment of a patient with CHF/COPD or is involved much too late in the terminal phase of life.<br>Many participants would welcome a protocol or a guideline on how to deal with the last hours of life of patient.<br>Word "palliative" is a bad word that results in undesirable confrontation with both patients and their families (supportive care)	
**77**	Udo et al 2018	Professionals	Individual interview	Old patients	The participants reported that incidents with a positive impact on palliative care situations included successful cooperation with both the patient's family members and colleagues.<br>Presence and provision of psychosocial support was reported as important factor to providing appropriate attention.<br>The care environment as an important factor in palliative care: Participants noted that positive incidents involved the discovery of how different environmental factors, such as a painting or photography, could awaken the dying person's memories and thus initiate a deeper dialogue. A noisy environment and a lack of peace and quiet contributed to the unease and worry of the dying person.	Participants were intentionally selected and were a homogeneous group; all of them were women born in a Scandinavian country. Data are limited to two municipalities in central Sweden
**78**	Wallerstedt et al 2018	Professionals	Focus group or similar	-	Challenge to communicate transitions:<br>Participants' perceptions of palliative care were also connected to how and when the transition to palliative care was communicated.<br>Need of interprofessional collaboration:<br>Interprofessional collaboration was described as important in palliative care to increase the ill person's well-being and to make the death as easy as possible. The participants noted that the care delivered to the ill person should be characterized by preserving dignity, which is facilitated if everybody is working	The use of multiple moderators in the study may have jeopardized the reliability of the results. The few numbers of participating physicians.

					in the same direction. However, it was said that this collaboration in palliative care could be improved using proactive, diverse professional knowledge earlier in the process.	
**79**	Pennbrant et al 2020 Sweden	Professionals	Individual interview	only 'older patients' is mentioned	Need for specialized palliative care for older people with dementia.<br>Specialized knowledge, experience and skills when working so close to death are needed.<br>Difficulties in meeting the needs of an elderly patient with dementia triggered feelings of stress and inadequacy due to his lack of specialized knowledge.<br>Need for teamwork to provide holistic care (doctors, nurses, occupational therapists, physical therapists and nursing assistants).<br>Need for time to know the patient's needs, determine itineraries and evaluate symptoms.<br>Need for communication and continuous contact with patients' families.	Small number of nurses included in this study reduces its transferability to other healthcare settings
**80**	Carvalho et al 2018	Professionals	Individual interview	-	Discoordination of the health care network, lack of resources for the provision of safe care, absence of professional training and influences of culture and the hospital-care centered model in palliative care in primary health care.	The study was conducted in only one Danish municipality, albeit large, which may limit the scope of the findings.<br>Empirical data from three specific professional groups analyzed as a single group can be considered a limitation.
**81**	Sheng-Yu et al 2017	Professionals	Individual interview, questionnaire/s or survey	-	Excessive workload without enough time and lack of support and teamwork from psychosocial care professionals<br>Lack of standardized ways to provide psychosocial care.<br>Interference from patients’ physical condition, e.g., symptoms, too tired, delirium.<br>Lack of consensus in medical team, e.g., ways to provide psychosocial care.	Sampling of participants for the quantitative survey was conducted via the Internet; therefore, they may not be representative of the entire population.

					Lack of skills or abilities in PC.<br>Lack of suitable indices to ensure effectiveness<br>Worries about saying something wrong that hurts patients and families<br>Need of tools to assess the psychosocial status and needs of patients and their families and training in communication skills, truth-telling and psychosocial care.<br>Support needs from psychosocial care professionals (social worker, clinical/counselling psychologist, chaplains/spiritual care professionals, other alternative therapy professionals, psychiatric physicians)	
**82**	Lenherr et al 2012	Professionals	Individual interview	-	Interviewees expressed a considerable willingness to talk about death and care with patients nearing the end of life, but expressed some obstacles to address it such as lack of time and/or privacy to address these issues; some cited personal reasons that prevented them from talking about death such as feeling confronted with their own mortality; they also mentioned resistance, aversion and refusal on the part of patients to discuss these issues with them; and the barrier that represents the cognitive state of some patients to be able to address the issue of care at the end of life.	The sample size is too small to make any general conclusions. Another limitation is the fact that the data were only collected in geriatric hospitals.
**83**	Bolt et al 2016	Patients, Relatives or informal caregivers, Professionals	Questionnaire/s or survey	23-88	The cases of appropriate care were diverse, but all involved care in (one or more of) five dimensions; supportive care, treatment decisions, location, the role of the patient's wish and communication.<br>Concern about situations of overtreatment and lack communication with professionals.<br>Desire to be involved in treatment decisions	Selection bias, participants were not randomly sampled.<br>Recall bias might also have led to some distortion.<br>Use of retrospective data.

**84**	Bijnsdorp et al 2021	Caregivers	Individual interview	only 'older patients' is mentioned	Worries about combining work and care.<br>Understanding from supervisors and colleagues at work, having autonomy at work, flexibility in working hours and being able to work remotely.<br>Having short lines of communication with healthcare professionals would help family caregivers to make decisions effectively and plan ahead.<br>Combination of work and care as burdensome.<br>Communication with healthcare professionals<br>Shortage in professional healthcare for the care recipient (e.g. long waiting lists).<br>Problems included bureaucracy, lots of paperwork, unclear application procedures and files that went missing, which made it hard for caregivers to arrange the care that was needed.<br>Frustration in relation to illness progression.<br>Support in Grieving process	This study doesn't include male caregivers. The results of this study are from a single stakeholder perspective.
**85**	Midtbust et al. 2018	Professionals	Individual interview, focus group or similar	-	Lack of continuity was identified as a major threat to the palliative care of people with severe dementia in long-term care facilities.<br>Time pressure and the scarcity of resources was pointed as a barrier to provide appropriate attention.<br>Advanced care planning was highlighted as a facilitator for providing palliative care.<br>Extensive use of temporary staff among nurses and doctors and the relocation between the sheltered and long-term wards threaten the continuity in planning and providing palliative care.<br>Palliative care for residents with severe dementia demands knowledge and precise observations.<br>Security and calmness is necessary for residents with	Recruitment may have been influenced by the preferences of the management; they may have chosen informants that they thought were suitable, and other potential informants who might have added important information may have been excluded.

					dementia, but this can be difficult to provide in a ward where staff must run back and forth constantly	
**86**	Fryer et al 2016	Professionals	Focus group or similar	-	Need for professionals (healthcare assistants) to receive greater support to address the emotional and practical aspects. A preference for education delivered through peer support was identified. Professionals working in residential care for elderly patients do not have time to process grief when patients die, as one patient is replaced by another immediately.	Due to the cohort of women, the study cannot include the experiences of male HCAs.
**87**	Mousing et al 2017	Patients, Professionals	Individual interview, focus group or similar	Old patients	Nonawareness of PC needs in patients with COPD and vague understanding of PC.<br>Organizational barriers to a PC approach and lack of time and continuity in attention.<br>Difficulties to initiate difficult conversations with patients.<br>The thought that PC was offered to patients with cancer and that initiatives primarily concerned pain management.	Empirical data from three specific professional groups has been analyzed as one group may be considered a study limitation.
**88**	Chan et al 2022	Professionals	Review, survey (Delphi)		Including trained volunteers in care delivery.<br>Exploring the spiritual, religious, and existential concerns of residents and family members.<br>Promoting person-centred and holistic care.<br>Maintaining residents' dignity, privacy, and autonomy.<br>Including nutritional screening in the initial assessment.<br>Formulate and regularly review nutrition and hydration care plan.<br>Managing the prescription of controlled drugs according to the local laws.	Study focused on the Hong Kong context, non-generalizable results. The response rate for the Delphi survey is relatively low and could not achieve Sumsion's standard
**89**	Barclay et al. 2011	Patients, Professionals	Review	60 and over	Lack of understanding of heart failure by patients.<br>Professionals worry about not being able to provide an accurate prognosis and feel they lack the skills needed for good communication with patients.<br>Patients fear discussing EoL care as it generates anxiety and loss of hope.	The smaller number of quantitative studies were given a lower weight of evidence, being retrospective or limited data being available from routine sources such as medical records.

					Professionals time pressures are seen by patients and clinicians as limiting the potential for conversations. Both patients and clinicians wait for the other to open up EOLC conversations which makes it difficult to start these conversations.	
**90**	De Witt Jansen et al. 2017	Professionals	Individual interview	-	Difficulty diagnosing due to the presence of neuropsychiatric symptoms (patients with dementia) that can result in inappropriate treatments. The profiles of patients with complex comorbidity and neurodegenerative diseases present difficulties in the prescription of drugs and treatments. Drug prescribing and symptom control is a challenge for the profile of patients with advanced dementia as there is no possibility of engaging in a discussion with patients. Care for patients with dementia requires the involvement of families and health professionals in all disciplines. Training needs through workshops to provide optimal care to this profile of patients was pointed as a need for professionals.	The sampling approach may have resulted in a biased sample of physicians with interest or previous experience in participating in research, who felt comfortable talking about professional challenges.
**91**	Hennings et al. 2010	Relatives or informal caregivers	Review	-	Family carers felt that they lacked knowledge of the dying process, they wanted communication, information and support in their decision-making, but they perceived professionals as lacking preparation and education for their role in guiding and supporting them in their decision making. Caregivers wanted to participate in the decision-making process and wanted continuous contact and communication with professionals, especially in EoL processes.	Not reported
**92**	Perone et al 2018	Patients	Individual interview	The sample contains more than 50% of	Patients expressed the following needs; lack of autonomy in daily activities, depression and loneliness, especially people with less mobility who perceived social isolation, dissatisfaction with pain control and symptom management,	Possibility of selection bias of participants

				patients over 65	were concerned about being a burden on families, sometimes felt that they had been treated without dignity and highlighted the lack of information. They also noted the lack of access to paid caregivers, home care, physical therapy, wheelchairs or diapers.	
**93**	Raymond et al 2012	Patients, Relatives or informal caregivers, Professionals	Review	only 'older patients' is mentioned	The importance of keeping the person with dementia in a stable care environment was highlighted by avoiding repeated moves from one family member's home to another's, and then to a residence. Family carers expressed frustration that they were not prepared for the end-stages of the dementia. Advanced planning for end of life was pointed as a need. Health professionals manifest a Lack of awareness of the person with dementia's. wishes among options and preferences should be discussed with the person and their carers while the person with dementia is able. Importance of religion for some people with dementia and for some carers who drew emotional strength from religious faith when witnessing the terminal stages.	This search was confined to electronic databases and focused on papers in peer-reviewed journals.
**94**	Hill et al. 2016	Patients, Relatives or Informal caregivers	Q-methodology (combination of qualitative and quantitative techniques)	Mean age 72.88 years	There are multiple perspectives of good EoL care for people with dementia; and thus, a 'one-size-fits-all' approach to planning is not appropriate to accommodate individual needs. Compassionate care is essential and incorporates respect for the person with dementia as an individual. Family members should have some involvement in medical decisions about the person with dementia together with health care staff. Comfort and safety of the person with dementia at the end of their life is crucial, for instance, receiving care in the same location to favor familiarity with surroundings, people and facilities and, thus, minimize distress.	The sample of participants with dementia was relatively small, findings may not be generalizable.

**95**	Montamedi et al 2021	Patients and caregivers	Review	Older patients (+60)	Effective communication between clinicians and patients/ caregivers. Accessible and frequent communication. Communication that involves the whole family and multidisciplinary team. Clear, comprehensive, and consistent information about the patients' condition, diagnosis, treatment, and prognosis. Healthcare that values patient preferences and shared decision-making. Discuss and respect patient's preferences for EOL care. Models of care that support care at home for as long as possible. Healthcare services that meet patient expectations: Staff well trained in managing specific conditions and complex issues. Support for family/caregivers in dealing with EOL challenges (emotional support, services for caregivers, information on how to manage symptoms)	Searches were only conducted in one database, Medline. Potential for exclusion of some countries if studies were not published in English language, selection bias amongst participants.
**96**	Steindal et al 2020	Patients and professionals	Review	65 years or over	The articles do not provide a clear definition of spiritual needs. The need to feel connected to others, such as family and other important people, was highlighted. Muslim patients report a large number of religious needs, such as praying, reading religious texts, and attending religious services. Patients interviewed in the studies report a high number of unmet spiritual needs and a lack of spiritual attention. The articles describe a lack of knowledge, competence, and training to address spiritual (professional) needs. More training, guidance, and training is needed to activate spiritual care in health care.	There may be terms that we have not been able to identify and include in our search strategy. Only included studies in English, Nordic, Spanish, and Portuguese.
